# Multiple Cutaneous Angiosarcomas after Breast Conserving Surgery and Bilateral Adjuvant Radiotherapy: An Unusual Case and Review of the Literature

**DOI:** 10.1155/2014/413030

**Published:** 2014-03-05

**Authors:** Icro Meattini, Raffaella Santi, Daniele Scartoni, Irene Giacomelli, Carla De Luca Cardillo, Vieri Scotti, Donato Casella, Roberta Simoncini, Lorenzo Orzalesi, Jacopo Nori, Milena Paglierani, Lorenzo Livi

**Affiliations:** ^1^Department of Radiation-Oncology, University of Florence, Largo G.A. Brambilla 3, 50134 Florence, Italy; ^2^Division of Pathological Anatomy, University of Florence, Largo G.A. Brambilla 3, 50134 Florence, Italy; ^3^Department of Surgery, University of Florence, Largo G.A. Brambilla 3, 50134 Florence, Italy; ^4^Diagnostic Senology Unit, University of Florence, Largo G.A. Brambilla 3, 50134 Florence, Italy

## Abstract

Breast angiosarcomas (BAs) are rare but serious events that may arise after radiation exposure. Disease outcome is poor, with high risk of local and distant failure. Recurrences are frequent also after resection with negative margins. The spectrum of vascular proliferations associated with radiotherapy in the setting of breast cancer has expanded, including radiation-associated atypical vascular lesions (AVLs) of the breast skin as a rare, but well-recognized, entity. Although pursuing a benign behavior, AVLs have been regarded as possible precursors of postradiation BAs. We report an unusual case of a 71-year-old woman affected by well-differentiated bilateral cutaneous BA, diagnosed 1.9 years after adjuvant RT for synchronous bilateral breast cancer. Whole-life clinical followup is of crucial importance in breast cancer patients.

## 1. Introduction

Angiosarcomas (ASs) are rare malignant tumors that arise from endothelial cells lining vascular channels [1, 2]. They account for less than 1% of all soft tissue sarcomas and occur in all organs of the body. Approximately 8% of ASs arise in the breast [3]. Primary breast angiosarcomas (BAs) most commonly affect women aged 20 to 40 years without a recognized associated factor [4].

Secondary BAs are usually found in older women at the site of radiotherapy (RT) for breast cancer (BC). They typically involve the dermis and present with skin changes that can easily be misinterpreted even with benign conditions such infection [5].

Neoplastic events attributed to RT in the context of BC are rare. Such occurrences are largely restricted to possible secondary lung and BC, osteosarcomas, malignant fibrous histiocytomas, and fibrosarcomas [5–8].

Postradiation BAs are defined by three characteristics: location in the field of radiation, latency of years after RT, and histologic distinction from the primary neoplasm [9]. The latency period, or interval between RT and the diagnosis of BA, ranges from 3 to 12 years, with most tumors occurring within 6 years after RT [10].

In recent years, the spectrum of vascular proliferations associated with RT in the setting of BC has expanded, including radiation-associated atypical vascular lesions (AVLs) of the breast skin as a rare, but well-recognized, entity [9, 11]. Since clinic and histologic overlap with well-differentiated BA is likely, AVLs represent a diagnostic and therapeutic challenge. Moreover, although pursuing a benign behavior, AVLs have been regarded as possible precursors of postradiation BAs [12].

We report an unusual case of a 71-year-old woman affected by well-differentiated bilateral cutaneous BAs, diagnosed 1.9 years after adjuvant RT for synchronous bilateral BC.

## 2. Case Report

In October 2009, due to a swelling in the central superior quadrant of the right breast, discovered by self-palpation, the patient underwent mammography showing a 20 mm opacity diagnosed as invasive BC at biopsy. The patient was subjected to wide excision of the lesion, biopsy of two sentinel lymph nodes, and contralateral reconstructive surgery. Histological examination showed a multifocal invasive lobular BC (classic and alveolar type), nuclear grade 2 with pathologic staging pT1cN0. Estrogen receptors and progesterone receptors were positive in 90% and 70% of neoplastic cells, respectively; Ki-67 proliferative index was <5% and HER2 status was negative.

On the left breast, pathology showed a ductal carcinoma in situ (solid and cribriform type), nuclear grade 2. Estrogen receptors and progesterone receptors were positive in 100% and 80% of neoplastic cells, respectively. Postoperative clinicoradiologic staging was negative. Hormonal therapy with letrozole was started.

Both breasts were irradiated. Right breast received a total dose of 60 Gy (50 Gy on the whole breast plus a boost of 10 Gy on the tumor bed) and left breast received a total dose of 50 Gy, with conventional fractions of 2 Gy/day. Computed tomography-based simulation (Big Bore Oncology, Philips, Andover, MA, USA) was performed; a three-dimensional RT planning Pinnacle system (Philips Medical System, Bothell, WA, USA) was used. Both breasts were irradiated with two tangential photon beams while boost was performed with electron beams with energy of 12 MeV. Acute side effects were scored according to the Common Toxicity Criteria Adverse Events (CTCAE, version 4). The treatment was well tolerated, with only bilateral breast erythema G1 and asthenia G1.

**Figure 1:**
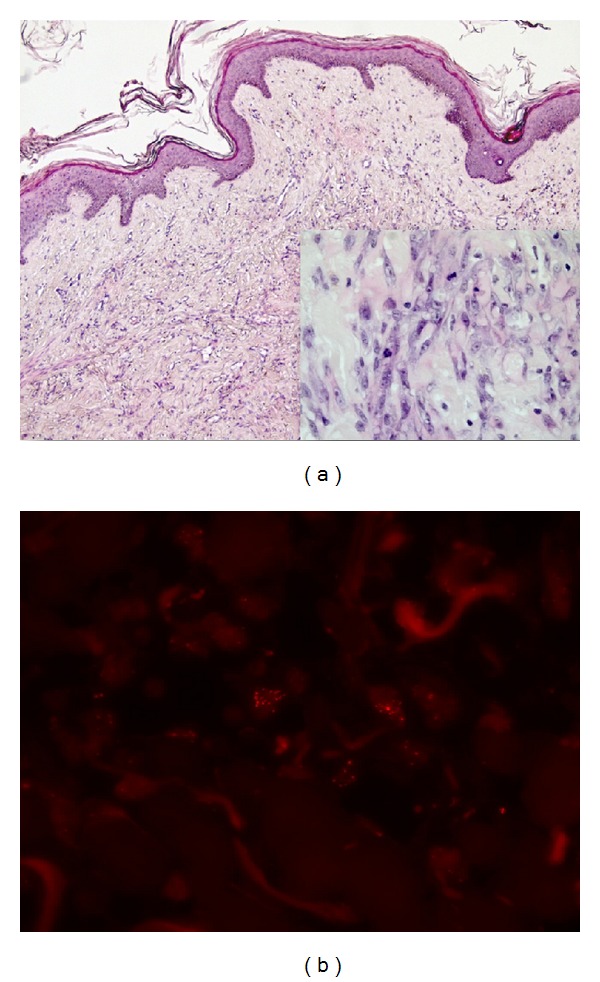
Reexcision of postradiation AVL of the breast skin one month after initial biopsy. Capillary vessels were lined by inconspicuous endothelial cells and randomly arranged throughout the dermis ((a); H&E, ×10). At the periphery of the lesion, a small focus displayed vascular structures with frank cytological atypia and mitotic figures, consistent with well-differentiated post-RT cutaneous BA (*inset*; H&E, ×40). MYC amplification at FISH analysis strongly supported the diagnosis of AS *versus* AVL (b). In order to detect copy number abnormalities of MYC oncogene (located at 8q24,12-q24.13), the LSI C-MYC SpectrumOrange Probe (Abbott Molecular Inc., Abbott Park, IL, USA) was used. The fluorescent signals were evaluated under an epifluorescence microscope (DMRD, Leica Mikrosystems Vertrieb GmbH, Germany), using a HBO 100 W mercury arc lamp and the appropriate single band filters (orange and blue) for the two fluorescence signals.

Regular follow-up visits were performed with annual bilateral mammography and breast ultrasound. In March 2012, at 1.9 years from RT, a small discolored area around the right surgical scar was detected; biopsy showed a radiation-associated AVL. Subsequently, the patient underwent wide excision with histologic diagnosis of cutaneous well-differentiated BA, arising as a small focus on a radiation-associated AVL (Figure 1(a)). Molecular analysis performed by fluorescence in situ hybridization (FISH) demonstrated the presence of MYC [13] amplification (Figure 1(b)). Surgical margins were negative.

**Figure 2:**
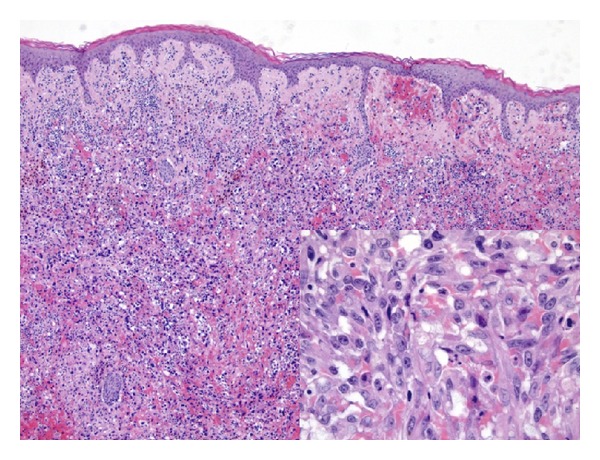
Surgical specimen displaying well-differentiated AS which arose in AVL (H&E, ×10; *inset*, H&E, ×40).

On January 2013, at 2.7 years from RT, a lesion appeared on the right breast; therefore patient underwent bilateral mastectomy. The final histology report showed bilateral mild differentiated cutaneous BAs in both breasts, diffusely infiltrating adipose tissue, and dermohypodermic stroma, with bilateral nipple-areola complex involvement (Figure 2).

Patient showed metastatic disease at restaging instrumental tests (multiple lung metastases) and started systemic therapy. At time of writing, 23 months since diagnosis of first BA, patient showed partial response to chemotherapy (endovenous docetaxel and gemcitabine, 6 cycles) and started hormonal therapy (letrozole).

## 3. Discussion

The development of malignant tumors after exposure to radiation is a well-recognized event. As a serious complication following therapeutic RT, the development of a sarcoma is a rare event [14]. The first reported case of BA arising within the skin overlying an irradiated breast was published in 1981 [15]. According to a large overview, BAs make up 15% of RT-related sarcomas [16].

Despite the apparent correlation between reports of secondary BAs and the increasing use of breast-conserving therapy, a true etiologic effect from RT has been difficult to establish. Although the preserved breast is the most common site of soft tissue sarcoma in patients receiving RT for BC, secondary BAs may also occur out of field [17].

Lymphedema has been implicated as a potential causative factor in the development of BA [18]. It has been suggested that the proliferation of lymphatic channels observed in patients with chronic lymphedema is mediated by growth factors that enhance the process of malignant transformation [4]. Chronic lymphedema may also produce a privileged site with reduced immunologic surveillance [1, 19]. Furthermore, exposure to RT may compound the risk of malignant transformation by its mutagenic effect and by causing lymphosclerosis, which exacerbates the obstruction associated with lymphadenectomy.

Compared to sporadic BAs, which usually arise in the parenchyma, RT-related BA is usually cutaneous; clinically, it presents as an erythematous plaque, patch, or nodules, often with edema, and evokes a differential diagnosis that includes inflammatory carcinoma or an infectious etiology [20]. Overall the prognosis for patients with RT-associated BA is poor, with high rates of local and distant recurrence [9, 17]. Vorburger et al. [21] showed a median disease-free survival (DFS) for patients with primary BAs of 6.5 years and a 5-year DFS rate of 56%.

Despite the high probability of local disease recurrence, the mainstay of treatment is a simple mastectomy or a wide local excision. A review reported published cases with at least 1 year of followup and showed that around 70% of patients developed BA recurrences within 1 year from surgery (Table 1). Even with negative margins through radical salvage surgery by simple mastectomy, radical mastectomy, or chest wall resections, additional local tumor recurrences are common [18].

Our unusual case is an example of how skin BAs after RT, although rare, are increasing phenomena. This case differs from the classic postradiation BA presentation reported in the literature. There are no risk factors which may explain this relatively short latency period (1.9 years); there is also no evidence of lymphedema.

Our report emphasizes the difficulty in differentiating well-differentiated BAs from AVLs on both the clinic and histologic findings [14]. Moreover, since AVLs are known to have a shorter interval from RT as compared to AS, postradiation AVL was the expected histological diagnosis. In the context of postradiation, histopathologic characteristics of vascular proliferations should be interpreted with extreme caution. As in this case, anastomosing small vessels exhibiting marked endothelial atypia and the presence of mitotic figures should favor the diagnosis of post-RT well-differentiated AS. In the differential diagnosis between these entities additional help comes from FISH analysis demonstrating MYC amplification, which is present in post-RT ASs but not in AVLs [13].

## 4. Conclusions

Postradiation BAs represent a rare but serious occurrence. Latency between RT and BA diagnosis may vary and may be influenced by several predisposing and treatment factors. Recurrences are frequent also after resection with negative surgical margins. Atypical vascular lesions require careful evaluation to rule out BAs. Clinical and instrumental followup is of crucial importance for these patients.

## Figures and Tables

**Table 1:** Major published studies concerning angiosarcomas of the breast after radiation.

Study	Age (yrs)	Time to BA (mo)	Surgery	Surgical margins	Nuclear grade	Time to first recurrence (mo)	Site of first recurrence	Treatment at first recurrence	Followup
Chahin et al., 2001 [22]	76	11	SM	Negative	High	3	Bilateral BA	None	Died 2 mo later
Feigenberg et al., 2002 [23]	72	77	SM	Negative	Low	2	Surgical scar	RT (50 Gy)	NED (39 mo after RT)
Feigenberg et al., 2002 [23]	73	66	SM	Close	High	1	Surgical scar	RT + WLE	NED (38 mo after RT)
Feigenberg et al., 2002 [23]	76	56	SM	Positive	Low	1.5	Surgical scar and flap	RT	NED (22 mo after RT)
Hildebrandt et al., 2001 [24]	79	66	SM	Negative	Low	4	Local recurrence	WLE	Alive with disease (23 mo)
Majeski et al., 2000 [19]	73	63	SM	Negative	—	26	Local recurrence	WLE	NED
Mills et al., 2002 [25]	77	96	SM	Negative	High	—	None	—	NED (14 mo)
Polgár et al., 2001 [26]	71	72	WLE	Negative	Int	3	Local recurrence	RM	NED (36 mo)
Sener et al., 2001 [27]	73	132	SM	Negative	High	3	Bilateral BA	RT	Dead (9 mo)
Solin et al., 2001 [28]	—	75	SM	—	—	—	None	—	NED (86 mo)
Vesoulis and Cunliffe, 2000 [29]	45	96	SM	—	—	—	—	—	No followup
Wang et al., 2009 [30]	87	108	SM	Negative	Int	9	Local recurrence	—	Alive with disease
Rao et al., 2003 [31]	59	168	RM	Negative	High	—	—	—	NED (41 mo)
Esler-Brauer et al., 2007 [32]	67	60	RM	Negative	Int	—	—	—	NED (45 mo)
*Meattini, 2014, present study *	*71 *	*23 *	*WLE *	*Negative *	*Low *	*9 *	*Bilateral BA *	*Bilateral RM *	*Alive with disease (23 mo) *

BC: breast cancer; BA: breast angiosarcomas; SM: simple mastectomy; RM: radical mastectomy; WLE: wide local excision; Int: intermediate; RT: radiation therapy; NED: no evidence of disease; mo: months; yrs: years.
